# The Burden of Caring for Children with Emotional or Conduct Disorders

**DOI:** 10.1155/2011/801203

**Published:** 2011-05-31

**Authors:** Howard Meltzer, Tamsin Ford, Robert Goodman, Panos Vostanis

**Affiliations:** ^1^Department of Health Sciences, University of Leicester, 22-28 Princess Road West, Leicester LE1 6TP, UK; ^2^Institute of Child Health, Peninsula Medical School, St Luke's Campus, Heavitree Road, Exeter EX2 8UT, UK; ^3^Department of Child and Adolescent Psychiatry, Institute of Psychiatry, Kings College London, De Cespigny Park, Denmark Hill, London SE5 8AF, UK; ^4^Greenwood Institute of Child Health, Westcotes House, Westcotes Drive, Leicester LE3 0QU, UK

## Abstract

*Introduction*. There is a paucity of evidence from epidemiological studies on the burden of children's emotional and conduct disorders on their parents. The main purpose of this study is to describe the problems experienced by parents of children with conduct and emotional disorders using data from a large national study on the mental health of children and young people in Great Britain. *Materials and Methods*. The Development and Well-Being Assessment and sections of the Child and Adolescent Burden Assessment were included in a nationally representative survey of the mental health of 10,438 children, aged 5–15, in Great Britain. *Results and Discussion*. Approximately half the parents of children with conduct disorder reported that they felt restricted in doing things socially with or without their children, embarrassed about their child's problems, and that these also made the relationship with their partner more strained. *Conclusions*. There is a growing need for research on the consequences of children mental disorders on families to increase the awareness of frontline workers on the burden to parents. Because parents feel embarrassed and stigmatized, they may hide their own feelings which may further exacerbate the situation.

## 1. Introduction

The terms, caregiver stress, caregiver strain, and caregiver burden have all been used to describe the needs, responsibilities, difficulties, and negative psychological effects of caring for a dependent relative [1, 2]. 

According to Brannan and Heflinger [3], caregiver strain has two dimensions: objective caregiver strain comprises the observable negative occurrences and constraints resulting directly from the child's problems (e.g., difficulties with neighbours and police, disrupted family relationships, financial strain, and interruption at work) and subjective caregiver strain which constitutes the caregiver's feelings towards those occurrences (e.g., stigma, guilt, anger, sadness, embarrassment, and worry). In brief, objective burden can be regarded as the observable disruption of aspects of the caregiver's life, whereas subjective burden is the extent to which the caregiver perceives care responsibilities to be stressful [4–6].

Parents and caregivers of children with emotional and behavioural disorders often experience significant burden associated with care of the child [2, 3, 6, 7]. These comprise financial burden, conflicts between family members, high irritability and overprotection in families, effect on family social life, interruption at work, fatigue, sadness and limitations on time, personal freedom, and privacy [2, 4, 8, 9].

Commonly, caregiver strain also has implications for children's entry into mental health services, engagement with practitioners, concordance with recommended interventions, and service use patterns when having treatment. 

As children with emotional or behavioural problems require more supervision and attention than a normal child would, parents tend to avoid public places such as cinemas, restaurant, shops, and public transport. Parents can also feel embarrassed and shamed by their child's behaviour when they visit relatives or friends. This results in reduced social contact. Adverse family interactions (parent-child, marital, and siblings) are also often linked to the child's behaviour [7].

In contrast to parents, other relatives caring for a child with emotional and behavioural disorder may not experience the same feeling of guilt or burden as parents [2]. However, kinship caregivers (e.g., grandparents, aunts, and siblings) have been reported to experience caregiver strain at levels similar to parents [4].

Using data from a large national study on the mental health of children and young people in Great Britain, the purpose of this study is to describe the problems experienced by parents of children with conduct and emotional disorders. These problems cover relationships with other family members, their social life, and expressions of stigma (embarrassment) and discrimination (avoidance). We examine the extent to which the distribution of the 10 items used to illustrate caregiver stress or burden are associated with biographic and sociodemographic characteristics of the child and parent. Among those parents who do express problems in caring for their children with emotional or conduct disorders, we examine their own contact with services in order to alleviate their stress.

## 2. Methods and Materials

### 2.1. Sample Design

The parents of each child under 16 living in the United Kingdom are entitled to receive child benefits unless the child is under the care of social services. The centralised computerized records from the Child Benefit Register (CBR) were used as a sampling frame to select children aged five to 15 throughout England, Wales, and Scotland. Using centralised records as a sampling frame was preferred for carrying out a postal sift of over 100,000 addresses and for sampling through school as it provides better coverage and is cost effective.

A total of 14,250 opt-out letters were dispatched by the Child Benefit Centre—30 letters for each of the 475 postal sectors. Nine hundred and thirty one of the sampled addresses (6.5%) opted out, and a further 790 addresses (5.5%) were found to be ineligible—the family had moved, and the child was deceased or had been placed in foster care. Among the cooperating families, almost all the parents and most of the children took part. Four out of five teachers returned their questionnaires, based on an initial mailout and one reminder letter.

Therefore, just over 12,500 addresses were allocated to around 300 interviewers. Information was collected on 83% of the 12,529 children eligible for interview from up to three sources resulting in at least some data for 10,438 children.

### 2.2. Measurement of Childhood Mental Disorders

The survey instrument used to produce the prevalence of clinically recognisable mental disorders among children was the Development and Well-Being Assessment (DAWBA). It was designed for use in the first national survey of child mental health in Great Britain. The DAWBA was constructed in order to combine some of the best features of structured and semistructured measures. This new structured interview was supplemented with open-ended questions. When definite symptoms were identified by the structured questions, interviewers used open-ended questions and supplementary prompts to get parents and young people aged 11 or over to describe the problems in their own words. An abbreviated form was mailed to a teacher nominated by the family as knowing the child well.

A case vignette approach was used for analysing the survey data, that is, using clinician ratings based on a review of all the information for each child—potentially from parent, child, and teacher. The case vignette approach was extensively tested among children who had been and those who had not been in contact with child mental health services in the prepilot and pilot phases of the survey [10]. Diagnoses were subsequently generated based on the ICD-10 research diagnostic criteria [11] using the information from all available informants.

In validation studies, the DAWBA provided excellent discrimination between children known to have mental disorders and those with no mental disorder [10]. There were high levels of agreement between diagnoses arising from the DAWBA data and from the case notes of children in contact with child psychiatrists. (Kendall's tau *b* = 0.47–0.70).

### 2.3. Measurement of Parental Burden or Stigma

Ten questions were chosen from the Child and Adolescent Burden Assessment [12] (later renamed the Child and Adolescent Impact Assessment) to represent both objective and subjective caregiver stress [13]. These questions were only asked of parents who indicated that they had significant problems with their children.

- Child's problems have kept parent from doing things socially with child—to a great extent, to some extent, or not at all.
- Child's problems disrupted parent's social and leisure activities—to a great extent, to some extent, or not at all.
- Child's difficulties have caused embarrassment—yes, no.
- Child's problems made relationship with partner—stronger, more strained, or made no difference.
- Child's problems made partner's relationship with other children—stronger, more difficult, or made no difference.
- Parent has felt disapproved of or avoided because of child's difficulties—yes, no.
- Child's problems made relationship with other children—stronger, more difficult, or made no difference.
- Child's problem put a strain on a previous relationship and was part of the reason the relationship broke up—to a great extent, to some extent, or not at all.
- Child's problems caused difficulties with parent's own relationships with friends—yes, no.
- Child's problems caused difficulties with other family members—yes, no.

A total burden and stigma score was calculated by summing any negative ratings across all 10 items—had difficulties to some or a great extent, made relationships more difficult or strained, or experienced embarrassment or avoidance.

### 2.4. Measurement of Socioeconomic Status

As questions on income have the highest rates of missing values in surveys in the UK, housing tenure was chosen as our measure indicative of socioeconomic status. Tenure was assessed by asking two standard questions: how the accommodation was occupied and who was the landlord. In terms of occupation, the response categories were owned outright, buying it with the help of a mortgage or loan, shared ownership, renting, living rent-free, and squatting. If the accommodation was rented, the precoded categories for types of landlord were local authority or council or New Town development or Scottish Homes, a housing association or co-operative or charitable trust, an employer (organisation) of a household member, another organisation, a relative or friend (before living there) of a household member, an employer (individual) of a household member, or an individual private landlord. For analysis, three types of housing tenure category were created: owned or buying with mortgage, privately renting (from a private individual or organisation), and social sector renting (from local authority, housing association, or charitable trusts).

## 3. Data Collection Procedure

Lay interviewers (approximately 300) regularly involved in the British Office for National Statistics surveys were used to collect the survey data. Special attempts were made to trace families whose addresses or names had changed because of various circumstances. Because of the need to collect accurate quantitative and qualitative data within the DAWBA, interviewer training emphasized the need to obtain respondents' descriptions of any problems and concerns in their own words, facilitating this with open-ended prompts and recording the answers verbatim.

Interviewers completed the face-to-face interview with the parent or main caregiver first—about 95% were mothers, and permission was subsequently sought to ask questions of the sampled child. Young people aged 11 or over had a private face-to-face interview and also completed a computed-assisted self-completion interview (CASI) directly on a laptop computer for more sensitive questions about violent behaviour, smoking, alcohol, and drug experiences. At the end of the interview, parents or carers were asked to nominate a teacher who knew the child well; the SDQ was subsequently mailed to this teacher. A reminder letter was sent to teachers who did not respond. 

## 4. Statistical Analyses

Our approach to the analysis of the survey data is initially to describe and compare by means of crosstabulations the burden and stigma experienced by parents according to the diagnosis of mental order—emotional disorders and conduct disorders. Because children can have co-occurring disorders, three categories were produced: emotional disorders, conduct disorders, and both emotional and conduct disorders. Significant differences are commented upon. Multiple linear regression analysis is used to examine the extent to which characteristics of the child (age and sex) and household characteristics (tenure) increase the likelihood of parents giving affirmative responses when presented with each burden or stigma item. We then return to crosstabulations to describe and compare parental reports of psychological distress associated with their child's mental disorder and what they did about such feelings. Finally, we use Chi-squared analysis to examine the relationship between the severity of caregiver burden and the likelihood of parents taking their children to see appropriate mental health services.

To improve the representativeness of the survey, a weighting procedure was applied to the data. The data were weighted (a) to take account of differential sampling of postal sectors by country within Great Britain, (b) to match the age/sex distribution of 5–15 year olds in the population at the time of the survey, and (c) to compensate for response variability by metropolitan and nonmetropolitan regions, that is, poorer response in inner cities.

## 5. Results

### 5.1. Parental Burden in Relation to Type of Disorder

There was a trend for the parents of children with emotional disorder to report the lowest level of burden on all items, with the parents of children with both conduct and emotional disorders reporting the highest level of burden. The parents of children with conduct disorder only reported levels of burden that were either intermediate or similar to the parents with co-occurring emotional and conduct disorder (Table 1).

### 5.2. Characteristics of the Child Associated with Caregiver Burden

Parents of girls with conduct disorder compared with parents of boys with conduct disorder were more likely to say that they experienced embarrassment caused by their child's behavioural difficulties (OR = 1.65, 1.01–2.68, *P* < .05). The converse relationship was found for parents of children with emotional disorders, and the odds of parents of daughters with emotional problems feeling embarrassed were half that of parents of boys (OR = 0.48, 0.26–0.88, *P* < .01). Parents of children and young people classified as having both emotional and conduct disorders did not appear to express different levels of embarrassment by sex nor did the sex of the child seem to be associated with any other expression of caregiver's burden.

On the other hand, the age of the child was related to two examples of caregiver stress, but this only occurred among children with emotional disorders.

Parents of younger children with emotional disorders were three times more likely to say that their child's problems caused difficulties with other family members (OR = 3.05, 1.52–6.12, *P* < .001). If the children had both conduct and emotional disorders, the parents of the younger children were more prone to report feeling disapproved of or avoided (2.42, 1.02–5.74, *P* < .05).

### 5.3. Characteristics of the Family Associated with Caregiver Burden

Socioeconomic status (as measured by housing tenure) was associated with different manifestations of parental burden depending on the type of the child's mental health problem. If the child just had a conduct disorder, parents living in social sector accommodation (provided by a local authority or housing association) were half as likely than owner-occupier parents to say their child's behaviour caused friction with other family members (OR = 0.52, 0.31–0.85, *P* < .01). Yet if the child just had an emotional order, private renters were far more likely to report feeling disapproved of than owner-occupier parents (OR = 2.33, 1.03–5.26, *P* < .05).

Parents living in social sector accommodation who had children with co-occurring conduct and emotional disorders were far more likely than owner occupiers to report that their leisure and social activities were disrupted (OR = 3.19, 1.27–8.01, *P* < .01) and that other people disapproved of them or avoided them (OR = 5.40, 1.96–14.90, *P* < .01).

## 6. Health of Burdened or Stigmatised Parents

When parents of children with conduct disorders were asked how their children's behavioural problems affected them, the vast majority, nine out of 10, said they were worried about their children, about three quarters said they were tired, and just less that two-thirds said it made them depressed. Just over a quarter said their child's problems made them physically ill (Table 2). There were similar trends in the pattern of responses to the items on burden, with parents of children with emotional disorders reporting less ill health than the parents of children with comorbid disorders, with the parents of children with conduct disorders being intermediate or similar in the level of ill health reported to parents of children with both emotional and conduct disorders. As Table 2 shows, there were substantial numbers of these parents reporting ill health. Between a quarter (ED only) and a half (ED and CD) actually went to their GP for help for their distress, and about two-thirds of them were prescribed medication. (Table 3) Again the parents of children with comorbid emotional and behavioural disorders reported the highest rates of consulting primary care and prescription medication. The survey did not include questions asking for the name of the medication.

## 7. Use of Services

Feelings or experiences of caregiver stress appeared to stimulate the parent to do something to reduce the problem (accessing appropriate child services) as well as seeking help from their GP to cope with their own difficulties. The data in Table 4 suggest that the greater the range of caregiver stress experienced, the greater likelihood the parent sought out health and educational and welfare services for their children. Compared with parents who said they had no caregiver stress, those who reported five consequences or feelings of burden were twice as likely to contact primary health services, three times more likely to use specialist health services, and fives times more likely to seek help from social services for their child's difficulties.

## 8. Discussion

### 8.1. Comparison with Other Studies

The substantial levels of parental burden resulting from the care of children with mental disorders were also detected in a US study [1]. Using the Child and Adolescent Burden Assessment (CABA), Angold et al. found that a significant proportion of their study sample had at least one perceived burden [1]. In a study in China, Liu and colleagues found that most parents of mentally ill children experienced pressure in their life, and 97.9% of them had increased anxiety [6]. Moreover, over half of parents in their study indicated that their leisure time significantly decreased, and over a third of them reported that they were reluctant to invite friends into their house since their child had developed their problems. Parental feelings of burden and stigma seem to be evident across cultures.

### 8.2. Why Elevated Rates among Conduct Disorders?

Our finding that children with conduct disorders seem to provoke the most caregiver burden fits into the well-established view that parents of children with externalised disorder report significantly elevated levels of caregiver strain related to their child's disorder [2, 4, 8]. One explanation that has been put forward is that externalizing behaviour is disruptive and difficult to manage, makes the caregiver role more challenging, and clearly adds considerable stress on the caregiver and family [4].

Emotional disorders are less persistent than conduct disorders and may be less “visible,” which may explain the lower rates of parental burden among parents of children with anxiety and depressive disorders. It is also possible that having a child with an emotional disorder may be (or perceived to be) more likely to attract sympathy and support, while parents of children with challenging behaviour may be more likely to be blamed, to fear being blamed, or to blame themselves for their children's difficulties. Self-stigmatisation among adults with mental illness may have as great a negative impact on the individual as active discrimination [14]. However, the current study suggests that there are socioeconomic influences on the impact of burden among parents, with emotional disorders leading to greater burden among families living in more deprived circumstances and the reverse being true for conduct disorders.

### 8.3. Caregiver Burden by Biographic and Sociodemographic Factors

Whereas some previous studies did not find the child's gender or age to be predictors of caregiver stress [6, 8], this study found that more parental embarrassment resulted from “gender atypical” disorders, that is, girls with behavioural problems and boys with emotional problems. This indicates that internalizing and externalizing disorders should be considered separately in terms of caregiver's burden.

### 8.4. Caregiver Burden and Use of Mental Health Services

The strong direct relationship between caregiver strain/burden and the increased use of children's mental health services has been found in several studies [15]. Caregiver strain has been identified by a number of studies as the greatest predictor of service use [1, 8, 16], and a growing body of evidence indicates that caregiver stain is associated with increased child use of mental health service beyond what can be explained by severity of symptoms [4]. For instance, parental burden from child psychiatric disorders was a major cause for mental health service use in rural areas [1]. The presence of perceived burden was associated with at least a fivefold increase in the rate of specialised child metal health service use by children [1]. However, our study found a fivefold increase in contact with social workers.

### 8.5. Study Limitations

Although the participation rate of parents and young people in the survey was high, about a quarter of sampled households could not be contacted or refused. Parents who refused to take part or could not be contacted may have a higher rate of caregiver stress or burden. In addition, there is evidence from previous child psychiatric surveys that rates of childhood psychopathology are higher among nonrespondent families [17, 18], which lead to biased estimates of prevalence of childhood mental disorders. In addition, parents who feel stigmatized may be hesitant to respond to a survey about their child's behaviour. Even though the data were weighted for nonresponse, it is not possible to assess the magnitude and direction of potential bias in the resulting rates.

### 8.6. Implications for Future Research

It would have been desirable to ask the caregiver strain questions to all parents in the survey. The parents of most of the children who ended up with the diagnoses of conduct or emotional disorders were asked these questions, but a few cases were missed, namely, those where conduct disorders were diagnosed entirely on the basis of teacher or youth reports and parents did not express any concerns. It seems plausible that these parents would be likely to report low levels of care-giver strain although some might be in conflict with the child's school about the existence of problems in the school context that were not visible at home. We might therefore have underestimated the level of caregiver burden.

Children's perceptions of burden or problems would have provided a more comprehensive understanding of the impact of the child's difficulties at a family unit level. Parenting attitudes, rearing practices, and capacity were not measured; as all have shown to mediate parent and child mental health problems, they might also be important mediators or moderators of the level of strain experienced by parents.

Carrying out longitudinal research in the general population as well as with high-risk families and families attending clinical services will be important in establishing the value of parental burden or strain and its interaction with parenting capacity and response to interventions in predicting child mental health outcomes, or vice versa. A wider conceptualization and measurement of burden to include the perceptions of children, teachers, peers, and other carers would also contribute to planning community and specialist interventions.

Although all the parents who were directed to answer the question on “burden” reported that their children had problems of clinical severity, it would be interesting for future research to also focus on children with conduct and emotional disorders whose parents do not report significant burden or care and thus understand the differences from families who do so and to identify factors that promote resilience in dealing with distress.

## 9. Conclusions

### 9.1. Implications for Practice

As the number of children with mental illness is increasing, the numbers of children requiring care from relatives is also growing. Consequently there is an increasing demand to understand the needs of caregivers. Understanding their needs is crucially important for planning interventions and testing the value of programmes to support caregivers [2]. Since parental strain has a detrimental effect on parental health (more worry, depression, physical ill health, and increased smoking and drinking) which in turn may negatively impact on the child's mental health, it is important to reduce the caregivers' burden, so that parents can provide sufficient care and maintain their own healthy life [2]. Perception of burden and stigma is real for the parents and should not be ignored and needs to be taken into consideration in treatment plans. It is particularly important that practitioners are sensitive to the high levels of guilt and embarrassment that many parents feel in relation to any psychological difficulties experienced by their child. Feeling blamed by practitioners, whether real or imagined, is likely to impair the therapeutic relationship and may adversely impact on interventions designed to alleviate the child's or family's difficulties.

Clinicians need to take into account the mechanisms which underpin parental beliefs and narratives on the nature of burden and stigma. Caregiver stress and burden can be reduced through additional practical support or interventions, such as respite care for the parent rather than the child, enhancing social networks through parent support networks, and encouraging links with self-help organisations for children with similar difficulties. Interventions for the parents, in particular, if they suffer from mental health problems themselves and joint/family interventions which target the causes and impact of burden in the parent-child relationship may improve the situation. Interventions for the parents (parental education, behavioural strategies) may also minimise and prevent burden/impact of child behaviours.

### 9.2. Focus of Interventions

As it is well established that parental and child distress expressed through a range of mental health problems are interlinked and often underpinned by parenting difficulties, interventions need to focus on reducing such distress among both parents and children, as well as enhancing the quality of family relationships. Indeed, there is growing evidence on the effectiveness of parenting and family interventions at multiple levels, that is, universal community programmes, targeted interventions for high risk families, and those provided at secondary health care level [19, 20]. What the findings of this study illustrate is the importance of recognising, acknowledging, and addressing the burden experienced by caregivers of children with emotional or conduct disorders, in particular, their feelings of stigma, embarrassment, reduced quality of life, and poorer health.

More specifically, clinicians and other professionals involved should adopt a broad assessment framework, irrespective of their theoretical stance, to recognise parents' and families' impact of perceived or real burden of care, whether this is primary or secondary to children's mental health problems. Second, they could reduce or alleviate the practical consequences of burden through family support, respite care, or community activities, depending on their level of need and available resources. Finally, parents' perceptions and experiences could form the focus of the intervention and become integrated with behavioural or family systemic strategies.

## Figures and Tables

**Table 1:** Burden and stigma experienced by parents of children with conduct disorder and/or emotional disorders.

Burden and stigma items	CD only	ED only	CD and ED
	**%**	**%**	**% **
Child's problems kept parent from doing things socially with the child			
To a great extent	20.1	8.4	22.9
To some extent	32.5	23.4	32.0
Not at all	47.4	68.2	45.1

Child's problems disrupted parent's social and leisure activities			
To a great extent	15.8	10.6	24.0
To some extent	34.3	28.6	36.9
Not at all	49.8	60.9	39.1

Child's difficulties have caused embarrassment			
Yes	46.2	10.6	46.3
No	53.8	89.4	53.7

Child's problems made relationship with partner*			
Stronger	13.9	19.2	15.2
More strained	45.6	19.1	60.4
No Difference	40.5	61.8	24.4

Child's problems caused difficulties with other family members			
Yes	35.3	14.2	34.8
No	64.7	85.8	65.2

Child's problems made partner's relationship with other children more difficult*			
Stronger	3.6	16.5	6.1
More difficult	37.6	18.5	45.3
No difference	47.7	56.3	33.4
No other children	11.1	8.8	15.2

Parent has felt disapproved of or avoided because of the child's difficulties			
Yes	33.6	16.6	44.1
No	66.4	83.4	55.9

Child's problems made relationship with other children			
Stronger	6.4	10.7	10.5
More difficult	29.8	16.0	46.2
No difference	57.1	66.3	40.9
No other children	6.7	7.0	2.3

Child's problem put a strain on a previous relationship and was part of the reason the relationship broke up*			
To a great extent	10.4	5.9	17.0
To some extent	15.2	5.1	12.1
Not at all	63.3	79.8	64.2
No previous relationship	3.4	0.0	0.0
Problem started since relationship	7.7	9.2	6.6

Child's problems caused difficulties with parent's own relationships with friends			
Yes	17.7	8.6	31.3
No	82.3	91.4	68.7
*Base*	312	306	90

**Table 2:** Parental reports of psychological distress by the type of child's mental disorder.

Burden and stigma items	CD only	ED only	CD and ED
Would you say they have made you worried?			
To a great extent	53.1	30.5	57.4
To some extent	39.9	51.6	35.8
Not at all	7.0	17.9	6.9

Would you say they have made you tired?			
To a great extent	30.5	18.4	41.1
To some extent	45.9	37.6	37.5
Not at all	23.6	44.0	21.4

Would you say they have made you depressed?			
To a great extent	21.0	13.6	35.6
To some extent	41.5	31.9	46.7
Not at all	37.5	54.5	17.7
*Base*			

Would you say they have made you psychologically ill?			
To a great extent	9.9	8.0	19.6
To some extent	18.2	12.3	26.3
Not at all	71.9	79.8	54.1

*Base*	312	307	90

**Table 3:** Parent's behaviour having experienced burden and stigma by the type of child's mental disorder.

	CD only		ED only		CD and ED	
Parent's behaviour	%	*N*	%	*N*	%	*N *
Had seen doctor because felt difficulty coping with child	30.9	297	26.0	273	50.3	88
Prescribed medicine among those who had seen GP	59.1	92	66.8	71	75.3	45
Drank more (if drank any alcohol)	8.5	263	9.3	233	16.6	73
Smoked more (if a smoker)	49.3	224	34.9	199	50.5	66

**Table 4:** Use of services for child by number of burden and stigma items.

Services used	Number of burden or stigma items				
	0	1-2	3-4	5 or more	Chi-squared
	**%**	**%**	**%**	**%**	
Education (e.g., teacher, educational psychologist)	29.9	40.9	56.1	65.5	*X* ^2^ = 50.48, *df* = 3, *P* < .001
Primary healthcare (e.g., GP, health visitor)	25.5	32.2	40.5	52.7	*X* ^2^ = 29.69, *df* = 3, *P* < .001
Specialist healthcare (e.g., Paediatrician)	10.4	24.0	30.1	30.3	*X* ^2^ = 23.91, *df* = 3, *P* < .001
Social services (e.g., social worker)	7.9	12.5	22.0	41.8	*X* ^2^ = 70.15, *df* = 3, *P* < .001

*Base*	*164*	*208*	*173*	*165*	
